# Muscle quality and spine fractures

**DOI:** 10.1002/jcsm.12915

**Published:** 2022-01-24

**Authors:** Miaomiao Wang, Jingjing Liu, Xin Chen, Xinru Wang, Xiao Chen

**Affiliations:** ^1^ Department of Radiology The Affiliated Hospital of Nanjing University of Chinese Medicine Nanjing China; ^2^ The First Clinical institute Nanjing University of Chinese Medicine Nanjing China; ^3^ Department of Radiology Shanghai Sixth People's Hospital, Shanghai Jiaotong University Shanghai China

The association between sarcopenia or low muscle quality and bone health, such as osteoporosis and fractures has been studied.^1^, ^2^ However, controversial results were reported because of the differences in populations or definition of sarcopenia. We have recently read the interesting article entitled ‘Body composition and osteoporotic fracture using anthropometric prediction equations to assess muscle and fat masses’ which showed the association between predicated lean body mass index (pLBMI), appendicular skeletal muscle index (pASMI), and body fat mass index (pBFMI) and fractures.^3^ This prospective study observed 2350 and 6175 fractures in men and women during a 3 year follow‐up. They found that pASMI was associated with total osteoporotic fracture risk. Interestingly, they also found that high pASMI was related to a decrease of spine fracture in both men and women. To the best of our knowledge, few studies independently observed the role of muscle quality on vertebral fracture. These data indicated that intervention on muscle quality may be a potential preventive strategy for reducing the risk of vertebral fracture.

There may be a couple of critical issues that were not considered in their analysis. In the multivariate cox model, many confounders were adjusted, such as age and physical activity. However, bone mass or bone mineral density or status of osteoporosis at baseline which was one critical determinant for fracture was not included.^4^ It is still unknown whether muscle quality, pLBMI, or pASMI is an independent risk factor for total fracture or spine fracture in their study. In addition, fasting serum glucose was considered as a confounder. Diabetes may be a better choice than glucose level because some diabetic subjects may have normal glucose level when they take anti‐diabetes drugs.

We also performed a prospective study in a Chinese population who underwent computed tomography (CT) scanning for lung cancer screening and had none spine fractures during 2016–2019. They were followed up for 1–5 years (median 2 years). We identified 72 subjects (17 men and 55 women) with new vertebral compression fractures and 212 age and gender matched control subjects. Muscle area and density of erector spinae at intermediate level of thoracic spine 8, 10, and 12 were determined as biomarkers of muscle quality. The muscle density and area of subjects with fractures were significantly lower than those without fracture (*Figure* 1). Then, the muscle area and density were divided into three groups based on the interquartile range (<Q1, Q1–Q3, >Q3). The cumulative hazard of adjusted hazard ratio (adjusted for age, gender, body mass index, bone mass, diabetes, and renal functions) for muscle area was 0.58 [95% confidence interval (CI): 0.34–0.98] for Q1–Q3 (9.2–12.3 cm^2^) and was 0.20 (95%CI: 0.1–0.49) for Q3 (>12.3 cm^2^) compared with the first quartile (<9.2 cm^2^). Similar results were found in women. The cumulative hazard curves in total population and women were shown in *Figure* 2. The subjects with low muscle density (<36 Hounsfield unit, HU) also had higher risk of VCF (*Figure* 2C). However, it seemed that no significant difference was found between those subjects with muscle density of 36–48 HU and >48 HU. Moreover, if bone mass was added into the cox model, the association between muscle density and risk of fracture became no strong. Recent studies showed that muscle density may have better ability than hip areal bone mineral density in discriminating hip fracture^5^ and muscle density was also well associated with muscle strength.^6^ The association between muscle density and spine fractures needed further investigation.

**Figure 1:**
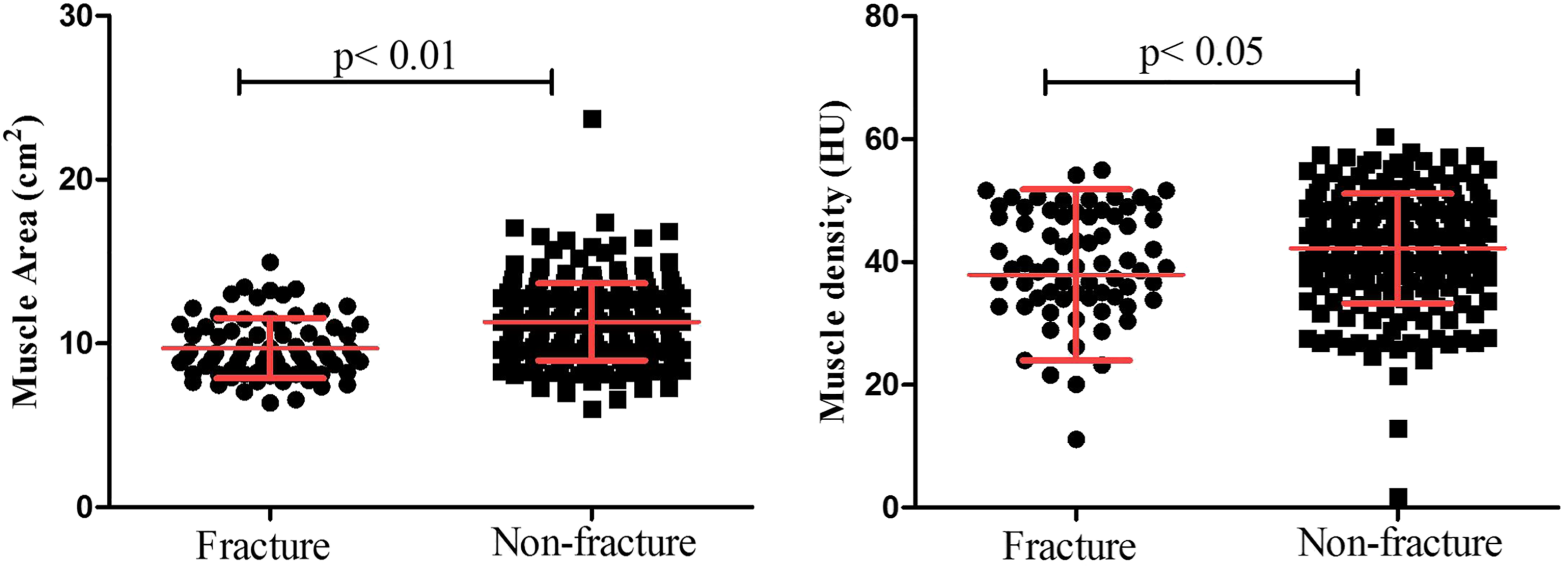
The muscle area and muscle density in subjects with and without vertebral compression fractures.

**Figure 2:**
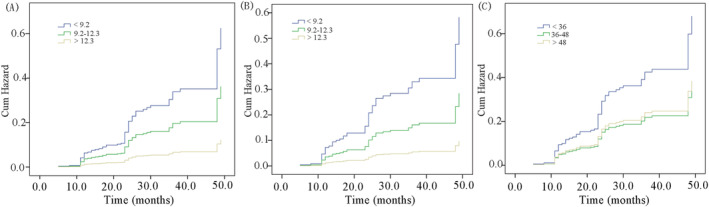
Cumulative hazards of vertebral compression fracture in total population (A) and women (B) stratified by muscle area and muscle density (C) during the follow‐up. Threshold of muscle area was set at <9.2 cm, 9.2–12.3 cm^2^, and >12.3 cm^2^. The threshold muscle density was set at <36 HU, 36–48 HU, >48 HU.

In conclusion, Hong *et al*. and our study showed that muscle quality is associated with risk of spine fracture. Our data also showed that this association was independent of bone mass. Intervention on muscle quality may be a helpful approach for reducing the risk of VCF. Excise on trunk muscle is welcome for the prevention of VCF in elders.

## Conflict of interest

None.

## Funding

This study was received supports from National Natural Science Foundation of China (No. 81773460, 81102148) and Educational Commission of Jiangsu Province (KYCX21_1632).
